# Genome‐wide effect of tetracycline, doxycycline and 4‐epidoxycycline on gene expression in *Saccharomyces cerevisiae*

**DOI:** 10.1002/yea.3515

**Published:** 2020-08-17

**Authors:** Guadalupe Sanchez, Samuel C. Linde, Joseph D. Coolon

**Affiliations:** ^1^ Department of Biology Wesleyan University Middletown Connecticut USA

**Keywords:** gene expression, RNA‐seq, tetracycline, yeast

## Abstract

Tetracycline (Tet) and derivative chemicals (e.g., doxycycline or Dox) have gained widespread recognition for their antibiotic properties since their introduction in the late 1970s, but recent work with these chemicals in the lab has shifted to include multiple techniques in all genetic model systems for the precise control of gene expression. The most widely used Tet‐modulated methodology is the Tet‐On/Tet‐Off gene expression system. Tet is generally considered to have effects specific to bacteria; therefore, it should have few off‐target effects when used in eukaryotic systems, and a previous study in the yeast *Saccharomyces cerevisiae* found that Dox had no effect on genome‐wide gene expression as measured by microarray. In contrast, another study found that the use of Dox in common cell lines and several model organisms led to mitonuclear protein imbalance, suggesting an inhibitory role of Dox in eukaryotic mitochondria. Recently, a new Dox derivative, 4‐epidoxycycline (4‐ED) was developed that was shown to have less off‐target consequences on mitochondrial health. To determine the best tetracycline family chemical to use for gene expression control in *S. cerevisiae*, we performed RNA sequencing (RNA‐seq) on yeast grown on standard medium compared with growth on media supplemented with Tet, Dox or 4‐ED. We found each caused dozens of genes to change expression, with Dox eliciting the greatest expression responses, suggesting that the specific tetracycline used in experiments should be tailored to the specific gene(s) of interest when using the Tet‐On/Tet‐Off system to reduce the consequences of confounding off‐target responses.

## 1 INTRODUCTION

Tetracycline family antibiotics were first discovered more than 70 years ago, and since their introduction for use in bacterial control in medical applications, they have gained widespread recognition for their potent antimicrobial properties (Grossman, 2016; Santiago‐Rodriguez et al., 2018). The tetracyclines are made up of a group of chemically similar compounds with a linear fused tetracyclic nucleus with functional groups attached along the periphery (Chopra & Roberts, 2001). The primary mechanism of action for their bacteriostatic properties is inhibition of protein synthesis via reversibly binding the 30S subunit of the bacterial ribosomal RNA thus blocking access of the aminoacyl tRNA to the A site of the ribosome (Chopra & Roberts, 2001; Das, Tenenbaum, & Berkhout, 2016). Inhibition of protein synthesis by tetracyclines is not restricted to bacterial species; they are also capable of inhibition of eukaryotic protein synthesis (Chatzispyrou, Held, Mouchiroud, Auwerx, & Houtkooper, 2015; Moullan et al., 2015), but because tetracyclines are actively taken up by most bacteria and not eukaryotic cells, their effects are generally assumed to be fairly specific to bacteria. This specificity has led to use of tetracyclines in eukaryotic experimental systems based on the inferred lack of off‐target effects, the most common use being control of transcription with the Tet‐On/Tet‐Off system (Gossen et al., 1995; Gossen & Bujard, 1992). This system is based on the bacterial Tet repressor protein (TetR) and Tet operator (TetO) DNA elements that control the Tn10 operon of *Escherichia coli* (Das et al., 2016) and involves the replacement of a gene's native promoter with one that can be controlled by the addition of a tetracycline allowing precise control of transcription activation or repression (Mnaimneh et al., 2004).

While this system was developed for use with the most basic member and namesake of the group tetracycline (Tet), its derivative doxycycline (Dox) is now more commonly used because it has increased stability (Agwuh & MacGowan, 2006; Honnorat‐Benabbou, Lebugle, Sallek, & Duffaut‐Lagarrigue, 2001) generating more consistent results in a very large number of studies to date. However, not much is known about the effects of Dox on global gene expression. Prior work conducted on yeast reported that the use of Dox does not lead to phenotypic effects nor does it have an effect on global gene expression (Wishart, Hayes, Wardleworth, Zhang, & Oliver, 2005). However, a more recent study showed that the use of Dox results in mitonuclear protein imbalance in commonly used cell types as well as in worms, flies, mice and plants (Moullan et al., 2015). Mitonuclear protein imbalance occurs when protein synthesis from mitochondrial DNA (mtDNA) does not match that of nuclear DNA (nDNA; Moullan et al., 2015). The mtDNA genome in yeast encodes 13 oxidative phosphorylation subunits that when exposed to Dox are not properly expressed, further affecting the stability of the complexes formed by these subunits (Jovaisaite, Mouchiroud, & Auwerx, 2014). As a result, this leads to the induction of the mitochondrial unfolded protein response (UPRmt) and causes the upregulation of mitochondrial chaperones and proteases (Jovaisaite et al., 2014). In turn, the UPRmt leads to a decrease in cellular respiration (Moullan et al., 2015). This can be detrimental to the organism's physiology but also may lead to a multitude of consequences in genome‐wide gene expression in response to these metabolic and physiological changes. Due to their bacterial ancestry, it is not surprising that tetracyclines have been shown to inhibit mitochondrial translation (Chatzispyrou et al., 2015). Recently, a newer Dox derivative 4‐epidoxycycline (4‐ED) was developed that was shown to have less off‐target consequences on mitochondrial function in mice (Eger et al., 2004). However, it remains unknown which common tetracycline compound (Tet, Dox or 4‐ED) is best suited for use in transcriptional control in laboratory experiments.

To determine the best tetracycline to use for gene expression control in the yeast *Saccharomyces cerevisiae*, we performed transcriptional profiling using RNA sequencing (RNA‐seq) on yeast grown on standard medium compared to growth on media supplemented with Tet, Dox or 4‐ED to identify genome‐wide changes in gene expression caused by exposure to these tetracyclines.

## 2 MATERIALS AND METHODS

### 2.1 Yeast strains and growth assay

**FIGURE 1:**
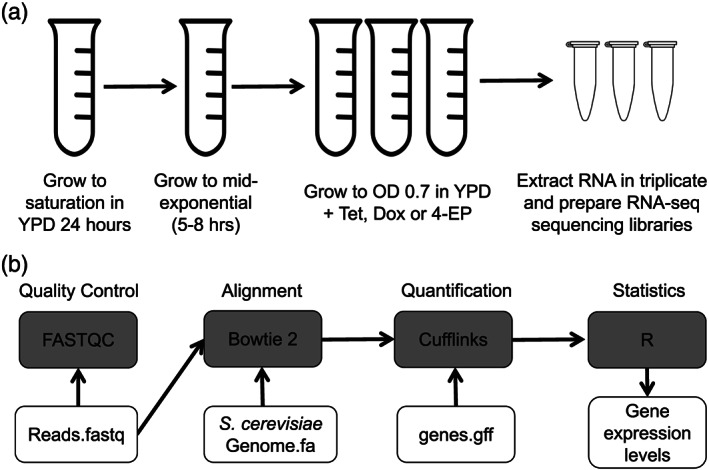
Experimental design and analysis pipeline. (a) The design of the experiment and sample collection is shown. (b) The bioinformatics pipeline used for RNA‐seq data processing and analysis is indicated

The yeast strain used for this study was the genetic background strain from the Yeast Tet‐Promoters Hughes Collection (yTCH) R1158 (URA3::CMV‐tTA MATa his3‐1 leu2‐0 met15‐0) which was derived from BY4741 (Mnaimneh et al., 2004). The strain was grown on YPD plates, and then, individual colonies were used to inoculate liquid YPD media. Liquid cultures were grown to saturation at 30°C and shaken at 200 RPM. From this culture, 100 μl was transferred to a new 5‐ml control YPD tube for 6 h to reach mid‐exponential growth‐based OD measurements and comparison with standard curves; 100 μl of this culture was then transferred to replicate tubes containing control YPD liquid medium or YPD media containing 1.5‐μg/ml Tet, Dox, or 4‐ED, and each was grown at 30°C and shaken at 200 RPM until the culture reached an OD_600_ between 0.6 and 0.8. Cells were then pelleted and used for RNA extraction. Cultures were grown in biological triplicate (four conditions × three replicates = 12 total samples) for downstream transcriptional profiling (Figure 1a). In each replicate, control YPD tubes grew at normal growth rates; however, cultures with tetracyclines added grew slowly and took longer to reach the target OD. We used 1.5 μg/ml for each tetracycline in this study because growth assays and titration experiments with Tet‐Off lines showed that there was no further effect of increases in concentration for each tetracycline beyond 1.5 μg/ml (data not shown).

### 2.2 RNA extraction, library preparation and RNA‐seq

Yeast pellets were incubated in a 100‐μl mixture containing 75 U of 20T lyticase at 30°C for 30 min. After incubation, total RNA was extracted using the SV total Isolation System (Promega) with a modified protocol (Coolon & Wittkopp, 2013). The recovered RNA was treated for 15 min with DNAse from the kit at room temperature. The mixture was then washed and eluted with 100 μl of nuclease‐free water and stored at −80°C. Prior to sequencing, RNA abundance and quality were confirmed with nanodrop spectrophotometer, Bioanalyzer and qubit. RNA samples were sent to the University of Michigan Sequencing Core Facility where mRNA selected bar‐coded libraries for each sample were constructed using the TruSeq library preparation kits and used in a single run of sequencing in a NextSeq‐500 with 76 cycles generating 438,947,573 reads for the 12 sequencing output files with an average of 36.5 million reads per sample (Table 1).

**TABLE 1:** Total number of mapped reads for RNA‐seq libraries

Sample	# reads	# mapping	% mapping
Control 1	37,276,053	36,560,731	98.1%
Control 2	40,352,284	39,439,313	97.7%
Control 3	41,194,014	40,314,390	97.9%
Dox 1	40,248,828	39,298,914	97.6%
Dox 2	31,976,734	31,251,336	97.7%
Dox 3	36,780,721	35,990,049	97.9%
Tet 1	32,333,060	31,676,304	97.9%
Tet 2	34,238,073	33,435,560	97.7%
Tet 3	31,962,937	31,128,128	97.4%
4‐ED 1	37,632,247	36,713,336	97.6%
4‐ED 2	38,027,360	37,232,697	97.9%
4‐ED 3	36,925,262	35,885,918	97.2%

### 2.3 RNA‐seq analysis and pipeline

After obtaining the sequence read files, an RNA‐seq pipeline (Figure 1b) was performed in Galaxy (Afgan et al., 2016) according to previously described methods (Lanno et al., 2017; Lanno et al., 2019). Briefly, FASTQC was used to ensure that the raw data was suitable for further statistical analysis (Andrews, 2010). The sequence reads were then mapped to the *S. cerevisiae* genome using Bowtie2 (Langmead & Salzberg, 2012; Langmead, Trapnell, Pop, & Salzberg, 2009) and the most recent genome file available at the time of analysis from ensembl: Saccharomyces_cerevisiae.R64‐1‐1.dna.toplevel.fa (Yates et al., 2016). Mapping was very successful with more than 97% of sequence reads mapping uniquely in each sample (Table 1). Gene expression quantification and differential gene expression analysis were carried out in Cuffdiff (Trapnell et al., 2010) using the *S. cerevisiae* genome file previously mentioned and the current gff3 file from ensemble: Saccharomyces_cerevisiae.R64‐1‐1.92.gff3 (Yates et al., 2016). R was used to perform all downstream analysis and generate visual graphics. Gene Ontology (GO) term enrichment was used on all annotated genes to identify enriched terms for biological processes, molecular function and cellular components (Ashburner et al., 2000; The Gene Ontology Consortium, 2014).

### 2.4 Data availability

All sequencing data generated for this manuscript are available at the Gene Expression Omnibus (GEO) under accession number GSE155989 (to be available at time of acceptance).

## 3 RESULTS

### 3.1 RNA‐seq identification of DEGs

**FIGURE 2:**
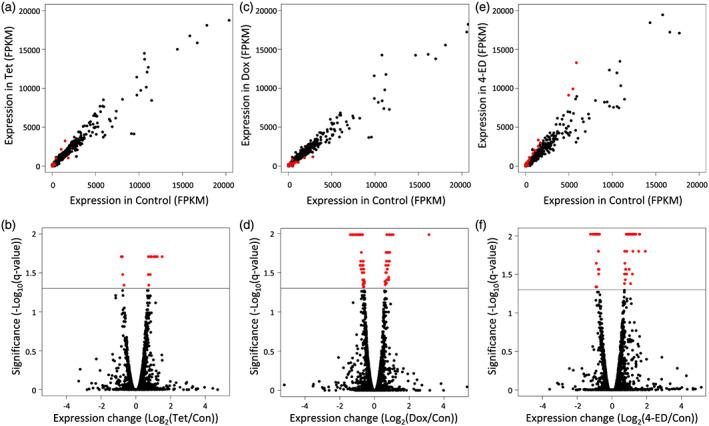
Identification of significantly expressed genes. (a,c,e) Scatterplots of all differentially expressed genes in *Saccharomyces cerevisiae* treated with 1.5 μg/ml of (a) Tet, (c) Dox and (e) 4‐ED in fragments per kilobase of transcript per million mapped reads (FPKM). (b,d,f) Volcano plots showing the magnitude of expression difference in control compared with treatments (b) Tet, (d) Dox and (f) 4‐ED on the *x*‐axis and −log_10_ transformed false discovery rate corrected *p* values (*q*‐values) on the *y*‐axis. (red = significant, black = non‐significant) [Colour figure can be viewed at wileyonlinelibrary.com]

**FIGURE 3:**
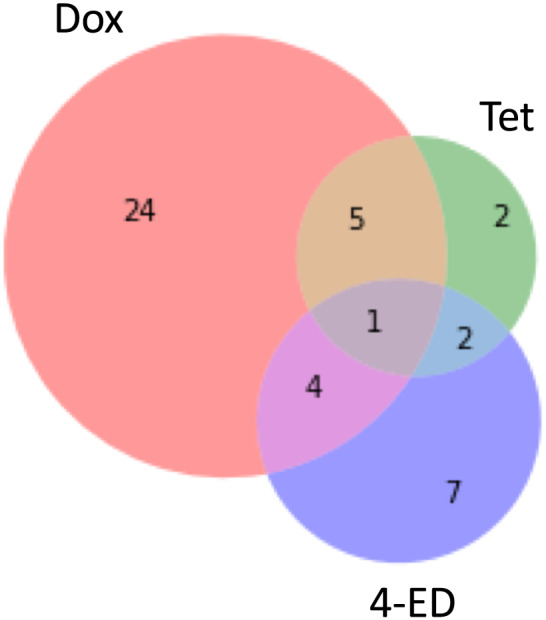
Overlap of genes identified as significantly differentially expressed in response to Tet, Dox and 4‐ED [Colour figure can be viewed at wileyonlinelibrary.com]

To determine which common tetracycline was best suited for control of yeast gene expression, we compared yeast grown in control media to yeast grown in media containing 1.5 μg/ml of Tet, Dox or 4‐ED. When yeast were grown in media containing Tet, 22 genes were significantly differentially expressed (*q* < 0.05) with 15 upregulated and seven downregulated (Figure 2a,b and Tables 2 and S1). When yeast were grown in media containing Dox, 83 genes were significantly differentially expressed with 27 upregulated and 56 downregulated (Figure 2c,d and Tables 2 and S2). Finally, when yeast were grown in media containing 4‐ED, 58 genes were significantly differentially expressed with 38 upregulated and 20 downregulated (Figure 2e,f and Tables 2 and S3). Interestingly, in Dox and 4‐ED treatments, significantly more genes were downregulated than upregulated (binomial exact test, *P*_*Dox*_ = 0.002, *P*_*4‐ED*_ = 0.01) whereas this was not observed for Tet treatment (binomial exact test, *P*_*Tet*_ = 0.13). When we compared the lists of differentially expressed gene (DEGs) for the three treatments, we found that there was very little overlap in identified genes that had annotations (Figure 3). In total, there were five unique genes identified as responsive to both Tet and Dox, four identified for Dox and 4‐ED, two for Tet and 4‐ED and one identified in all three treatments (Table 3; *SFC1*). Although only a few genes are found to overlap between identified gene sets, the amount observed is significantly greater than expected by chance in each pairwise comparison (Fisher's exact test, *P*_*Tet‐Dox*_ = 1.4 × 10^−7^, *P*_*Dox‐4‐ED*_ = 0.0005, *P*_*Tet‐4‐ED*_ = 0.0007).

**TABLE 2:** Differentially expressed genes (DEGs) identified responding to treatment with Tet, Dox or 4‐ED

Treatment	# DEG	# upregulated	# downregulated
Tet	22	15	7
Dox	83	27	56
4‐ED	58	38	20

**TABLE 3:** Overlap of genes identified as responding to Tet, Dox or 4‐ED treatments

Genes in all three	Genes in Dox/Tet	Genes in Dox/4‐ED	Genes in Tet/4‐ED
*SFC1*	*VID24*, *GIN4*, *SMC1*, *ASF1* and *KAR3*	*HOR2*, *CAR2*, *PDE3* and *CAR1*	*PCK1* and *FBP1*

### 3.2 Gene ontology term enrichment analysis for DEGs

To determine if there was enrichment for particular biological annotations for the sets of annotated genes identified as responding to the three tetracyclines tested, we performed GO enrichment analysis. We found that for the genes responsive to Tet, no GO terms were enriched for biological process, molecular function or cellular component terms. We found that for the genes responsive to Dox, several biological processes were enriched and one cellular component involved in DNA replication was enriched, including cellular response to DNA damage stimulus, double strand break repair (DSRB), cell cycle processes and terms associated with the replication fork (Table S4). Additionally, enrichment for biological processes involved in mitotic sister chromatin cohesion was observed. For genes identified as responsive to 4‐ED, GO term analysis showed enrichment in biological processes involved in the catabolic process of arginine and the metabolic processes of fructose and glucose (Table S5).

## 4 DISCUSSION

To determine the best tetracycline to use for gene expression control in *S. cerevisiae*, we performed RNA‐seq on yeast grown on standard medium compared with growth on media supplemented with Tet, Dox or 4‐ED to identify changes in gene expression caused by this exposure. We found that although the numbers of genes with response to Tet, Dox or 4‐ED are not large, there are dozens responding to each chemical. In fact, the results for Dox are in contrast with a prior report that found no genes responded to Dox using microarrays (Wishart et al., 2005). These contrasting results may be due to the differences in the methods used for gene expression analysis. It is generally accepted that RNA‐seq has a higher specificity, sensitivity and dynamic range and is therefore able to detect more DEGs including genes with very high or low expression than microarray‐based gene expression quantification (Rao et al., 2019). Another report found that the presence of Dox results in mitonuclear protein imbalance (Moullan et al., 2015). Yet our study found DEGs in the presence of Dox compared with the control that did not contain enrichment for any mitochondrial terms. Instead, various terms related to DNA replication were enriched for the DEGs. Interestingly, our results for 4‐ED showed that this chemical alters genes involved in arginine catabolism as well as those involved in glucose and fructose metabolism, two processes that require proper mitochondrial health. Furthermore, the genes identified overlap significantly between the different tetracycline derivatives suggesting a common mechanism of response. Additionally, GO term enrichment analysis suggested that in Dox and 4‐ED treatments, there was enrichment for genes with particular biological or molecular functions, suggesting a common biological and/or regulatory rationale for this response.

### 4.1 Effects of Dox on genes associated with DNA replication, cell cycle and mitosis

When *S. cerevisiae* was grown on medium containing Dox, various GO terms for DNA replication processes were enriched among the genes differentially expressed. Of particular interest were genes *CDC45*, *RNR1*, *RAD53*, *ECO1*, *POL1*, *POL2*, *SMC1* and *RTT107*, as they were all upregulated when yeast were treated with Dox and have GO terms for DNA replication and DSBR leading to enrichment of these GO terms. In yeast, *POL1* and *POL2* code for DNA polymerase I and II (Araki et al., 1992) and the upregulation of both *POL1* and *POL2* may suggest global increases in DNA replication. Upregulation of *POL1* and *POL2* alongside activation of *RAD53* suggests that there may be detrimental effects of the increases in DNA replication and *RAD53* may be stabilizing stalled or stressed replication forks during S phase (Szyjka et al., 2008), slowing down the DNA synthesis (Paulovich & Hartwell, 1995; Paulovich, Margulies, Garvik, & Hartwell, 1997) to increase survival (Branzei & Foiani, 2006). Genes with GO terms associated with mitotic sister chromatid cohesion and mitotic cell cycle were also differentially expressed when yeast were grown on medium containing Dox. Interestingly, all five genes associated with this enrichment were also upregulated (*RMI1*, *SMC1*, *ECO1*, *POL2* and *KAR3*), suggesting an increase in cell cycle and mitosis when yeast are exposed to Dox, similar to that observed for DNA replication genes. This is in contrast to the slowed growth rate we observe for yeast exposed to Dox in their media, and the disconnect between these processes requires further studies. All three of the major GO terms enriched point to upregulation of processes associated with the S phase of the cell cycle suggesting some mechanistic link between these identified genes. It is also possible that all the genes identified have a common regulatory element(s) that are all responding to the presence of Dox directly or indirectly and there may not be a specific functional relationship among the regulated genes. Either way, our findings suggest that use of Dox may not be ideal for studies focused on DNA replication, DNA repair, cell cycle, chromosome cohesion or any genes associated with or downstream of these processes.

### 4.2 Effects of 4‐ED on metabolic processes

When *S. cerevisiae* was grown on medium containing 4‐ED, various GO terms for arginine catabolism were enriched among the genes differentially expressed with both genes directly involved in arginine catabolism downregulated in the presence of 4‐ED (*CAR1* and *CAR2*). Changes in the expression of these genes could alter processes including protein synthesis as well as nitrogen utilization. Genes with GO terms associated with glucose and fructose metabolism were also differentially expressed when yeast were grown on medium containing 4‐ED. Among the four genes involved in glucose and/or fructose metabolism, two were downregulated in the presence of 4‐ED (*HXK2* and *PFK27*) and the other two were upregulated (*PCK1* and *FBP1*). Reductions in expression for key enzymes in metabolic processes including glucose and/or fructose metabolism could explain previous reports of reduced mitochondrial function in widespread organisms including mammalian cells (human and mouse), nematodes, fruit flies and plants (Moullan et al., 2015). The contribution of tetracycline‐induced transcriptional responses to mitochondrial dysfunction relative to changes in protein expression, function or other physiological changes remains an open question and will require future experiments to determine. Similar to that described above for Dox, it is possible that all the genes identified have a common regulatory element(s) that are all responding to the presence of 4‐ED directly or indirectly or there may be some specific functional relationship among the regulated genes. Regardless, our findings suggest that use of 4‐ED may not be ideal for studies focused on metabolism or downstream of these processes.

### 4.3 *SFC1* and its role in the yeast genome

*SFC1* is one of 35 members of the mitochondrial carrier family (Palmieri et al., 2000), acting as a succinate/fumarate mitochondrial transporter that is required to transport cytosolic succinate into the mitochondrial matrix in exchange for fumarate (Palmieri et al., 2000). The upregulation of *SFC1* in response to treatment with Tet, Dox and 4‐ED suggests an increase in the entry and exit of succinate and fumarate, respectively (Palmieri et al., 2000). Fumarate is a critical component of the gluconeogenic pathway which is important for proper cell growth in *S. cerevisiae* (Palmieri et al., 2000), suggesting that Dox, Tet and 4‐ED may be altering the growth of cells by inducing the increase in the expression of *SFC1.* Future experiments investigating mitochondrial transport with and without exposure to tetracyclines are warranted to demonstrate possible functional consequences of tetracycline‐induced gene expression changes in *SFC1.*

### 4.4 Similarity of tetracycline response across eukaryotes

In diverse eukaryotic organisms including mammals, insects, nematodes, plants (Moullan et al., 2015) and reported here in the fungus *S. cerevisiae*, exposure to tetracycline family chemicals causes detrimental effects on metabolism and mitochondrial function. The similarity of response across Eukaryota suggests an ancestral origin and could imply the underlying molecular mechanism of these effects is also shared. In general, tetracycline exposure elicits decreased respiration, metabolism and mitochondrial function. In those species tested for transcriptional response to tetracyclines, there appears to be common responses in genes from pathways likely involved in these processes including metabolic enzymes, mitochondrial transport, electron transport chain components and ATP synthesis (Moullan et al., 2015). The genes and pathways identified thus far are all excellent candidates for their contribution to disruption in mitochondrial function after tetracycline exposure. In addition to those that have potential effects specific to the mitochondria, induction of stress response genes appears to also occur across the eukaryotes tested thus far. Whether this is a direct response to tetracycline exposure or downstream of mitochondrial dysfunction is currently unknown and experiments focused on disentangling these effects warrant further study.

## 5 CONCLUSION

Due to the flexibility, precision and specificity of the changes that can be achieved using the Tet‐On/Tet‐Off system, it has become a powerful tool in research aiming to control gene expression in many model systems. Despite these major benefits, caution when using these chemicals to study gene function and expression is recommended. Recent reports have already suggested a potential negative impact of these antibiotics on the environment and health (Moullan et al., 2015) and have shown that Dox has effects on multiple diverse organisms and in many different types of cells and tissues (Luger et al., 2018; Moullan et al., 2015). Yeast are not to be excluded from this set of model organisms, as we show here that all three tested tetracyclines (Tet, Dox and 4‐ED) elicit changes in dozens of genes expression levels in *S. cerevisiae.* Although our results do not support the study that showed alterations in genes involved in mitochondrial translation, we observed other effects on distinct functional groups of genes including genes associated with the S phase of the cell cycle in response to Dox and metabolism in response to 4‐ED. Although we do not yet know the molecular mechanism by which these genes are changing expression, our data suggest great care should be taken using this system and the specific tetracycline chosen should be tailored for use in specific projects. Use of the tested tetracyclines (Tet, Dox, 4‐ED) for study of the genes identified herein as DEGs or genes involved in associated biological processes seems potentially problematic. That said, the majority of the genome does not respond to these chemicals suggesting they should still be available for widespread use in the testing of gene function as long as their limitations are first considered. Moving forward, we suggest that when using the yeast Tet‐On/Tet‐Off system for inferences about genome‐wide gene expression, controls similar to those performed here (comparing control to tetracycline exposure in background strains) should be included in experimental designs to ensure that off‐target effects are not the source of any observed effects on gene expression.

## CONFLICT OF INTEREST

The authors declare no conflicts of interest.

## Supporting information


**Table S1:** List of significantly differentally expressed genes upon exposure to 1.5 μL Tet
Table S2: List of significantly differentally expressed genes upon exposure to 1.5 μL Dox
Table S3: List of significantly differentally expressed genes upon exposure to 1.5 μL 4‐ED
Table S4: GO Term enrichment of significantly expressed genes when treated with Dox
Table S5: GO Term enrichment of significantly expressed genes when treated with 4‐ED
